# Identification of Potential Key Genes Associated With the Pathogenesis, Metastasis, and Prognosis of Triple-Negative Breast Cancer on the Basis of Integrated Bioinformatics Analysis

**DOI:** 10.3389/fonc.2020.00856

**Published:** 2020-06-12

**Authors:** Bin Zhao, Yali Xu, Yang Zhao, Songjie Shen, Qiang Sun

**Affiliations:** ^1^Department of Breast Surgery, Peking Union Medical College Hospital, Beijing, China; ^2^Department of Surgery, Peking Union Medical College Hospital, Beijing, China

**Keywords:** triple-negative breast cancer, bioinformatics, Gene Expression Omnibus, The Cancer Genome Atlas, SDC1, S100P

## Abstract

**Objective:** Breast cancer is the most common solid tumor affecting women and the second leading cause of cancer-related death worldwide, and triple-negative breast cancer (TNBC) is the most lethal subtype of breast cancer. We aimed to identify potential TNBC-specific therapeutic targets by performing an integrative analysis on previously published TNBC transcriptome microarray data.

**Methods:** Differentially expressed genes (DEGs) between TNBC and normal breast tissues were screened using six Gene Expression Omnibus (GEO) datasets, and DEGs between metastatic TNBC and non-metastatic TNBC were screened using one GEO dataset. Kyoto Encyclopedia of Genes and Genomes (KEGG) and Gene Ontology (GO) enrichment analyses were performed on the overlapping DEGs. The Cancer Genome Atlas (TCGA) TNBC data were used to identify candidate genes that were strongly associated with survival. Expression of the candidate genes in TNBC cell lines was blocked or augmented using a lentivirus system, and transwell assays were used to determine their effect on TNBC migration.

**Results:** Eight upregulated genes and nine downregulated genes were found to be differentially expressed both between TNBC and normal breast tissues and between metastatic TNBC and non-metastatic TNBC. Among them, S100P and SDC1 were identified as poor prognostic genes. Furthermore, compared with control cells, SDC1-overexpressing TNBC cells showed enhanced migration ability, whereas SDC1 knockdown markedly reduced the migration of TNBC cells.

**Conclusion:** Our study determined that S100P and SDC1 may be potential treatment targets as well as prognostic biomarkers of TNBC.

## Introduction

Although diagnosis, treatment, and survival have obviously improved, breast cancer remains the most common solid tumor affecting women and the second leading cause of cancer-related death in women worldwide (1). Triple-negative breast cancer (TNBC) is defined by the absence of estrogen receptor (ER) and progesterone receptor (PR) and the lack of overexpression of HER2 protein (2). TNBC accounts for ~13% of all breast cancers (3). TNBC is correlated with more aggressive tumor pathology, higher relapse rate, and poorer clinical prognosis than are other hormone receptor-positive subgroups (4). Although biomarkers and therapeutic targets used in breast cancer have made a great contribution to the improvement of the diagnosis and treatment of breast cancer, TNBC patients cannot benefit from the developed treatment strategies because of the lack of TNBC-specific therapeutic targets. Traditionally, the main therapeutic strategy for TNBC patients is a combination of radiotherapy and chemotherapy. Frequently, TNBC has a high sensitivity to chemotherapy, but it still has a high risk of recurrence and metastasis and poor overall survival (OS), which is called the “triple-negative paradox” (5). Therefore, novel therapeutic targets for these patients are urgently needed.

The application of microarray and high-throughput sequencing technologies in clinical research is becoming increasingly valuable, providing an efficient way to elucidate the alteration of critical genes in carcinogenesis and discover promising biomarkers for cancer diagnosis, treatment, and prognosis (6, 7). In recent years, integrated bioinformatics methods have been widely applied to cancer research owing to their advantages of overcoming the inconsistency caused by the variety of microarray platforms and the limitation of the small sample size (7–9).

In the present study, we detected the genes participating in both the carcinogenesis and metastasis of TNBCs using previously published TNBCs transcriptome microarray data, aiming to find potential TNBC-specific therapeutic targets.

## Materials and Methods

### Gene Expression Profile Data

Seven Gene Expression Omnibus (GEO) datasets (GSE65216, GSE31448, GSE45827, GSE61724, GSE36295, GSE37751, and GSE103091) were downloaded from the GEO (https://www.ncbi.nlm.nih.gov/geo/). GSE65216, GSE31448, and GSE45827 are based on the GPL570 platform (Affymetrix Human Genome U113 Plus 2.0 Array) and included 111 TNBC tissues and 26 normal breast tissues. GSE61724, GSE36295, and GSE37751 are based on the GPL6244 platform (HuGene-1_0-st Affymetrix Human Gene 1.0 ST Array) and included 41 TNBC tissues and 56 normal breast tissues. GSE103091 is based on the GPL570 platform and consists of 107 TNBC tissues, which contain 31 samples from patients with metastasis and 76 without metastasis. The characteristics of the samples from these datasets are shown in Table 1.

**Table 1:** Characteristics of the included datasets.

**GEO ID**	**Platform**	**Number of samples**		**Publication date**	**Institution**	**Country**
		**TNBC**	**Normal**			
GSE65216	GPL570	55	11	2015	Curie Institute	France
GSE31448	GPL570	74	31	2011	Paoli-Calmettes Institute	France
GSE45827	GPL570	41	11	2016	Curie Institute	France
GSE61724	GPL6244	16	4	2015	University of Newcastle	Australia
GSE36295	GPL6244	11	5	2014	King Abdulaziz University	Saudi Arabia
GSE37751	GPL6244	14	47	2013	National Cancer Institute	USA
GSE103091	GPL570	107	0	2017	ICO-UMGC	France

*GEO, Gene Expression Omnibus; TNBC, triple-negative breast cancer*.

### Differentially Expressed Gene Identification

The batch effect correction of the GSE datasets was conducted using the package “Sva” (version 3.29.1) of R language (version 3.5.1). Differentially expressed genes (DEGs) associated with the TNBC group and normal breast tissues group were analyzed using the “Limma” package (version 3.37.4). DEGs between TNBC with metastasis and without were obtained from the GEO2R online analysis tool. *P* < 0.01 and |FC (fold change)| ≥ 1.5 were set as the DEG cutoff criteria. Venn diagrams (http://bioinformatics.psb.ugent.be/webtools/Venn/) were generated to display the overlap of DEGs between the three datasets.

### Gene Ontology and Pathway Enrichment Analysis of Differentially Expressed Genes

The Database for Annotation, Visualization and Integrated Discovery (DAVID version 6.8) was used to perform Gene Ontology (GO) enrichment analysis and Kyoto Encyclopedia of Genes and Genomes (KEGG) pathway enrichment analysis to expound the biological processes (BPs), molecular functions (MFs), cellular components, and pathways associated with the overlapping DEGs.

### Survival Analysis

The clinical information and mRNA expression profiles of breast cancer patients were downloaded from The Cancer Genome Atlas (TCGA) database. After removal of patients without OS data and patients of molecular subtypes other than TNBC, 138 TNBC patients were selected for survival analysis. Candidate genes that were strongly associated with survival were identified through Kaplan–Meier survival analysis using the R package “survival” and “survminer.”

### Cell Culture

The human breast cancer cell line MDA-MB-231 was obtained from the Institute of Basic Medical Sciences of the Peking Union Medical College and Chinese Academy of Medical Sciences and cultured in Dulbecco's modified Eagle's medium (DMEM) supplemented with 10% fetal bovine serum (FBS). All cells were kept in a humidified atmosphere of 5% CO_2_ at 37°C.

### Cell Transduction

SDC1 overexpression and shRNA expression lentiviral particles and their negative controls were purchased from GenePharma (Shanghai, China). The sequences of the SDC1-shRNA were (forward) 5′-GGAGCAGGACTTCACCTTTGA-3′ and (reverse) 5′-TCAAAGGTGAAGTCCTGCTCC-3′. MDA-MB-231 cells were seeded at ~4 × 10^5^ per well in six-well plates and incubated for 24 h and then transduced with SDC1 overexpression or shRNA expression lentivirus or their control empty lentivirus for 48 h. Puromycin (Santa Cruz Biotechnology, Santa Cruz, CA, USA) was used to select these cells for 2 weeks. The efficiency of infection was assayed by quantitative PCR (qPCR).

### Real-Time PCR Assay

Total RNA was isolated from cultured cells using TRIzol reagent (Invitrogen, Carlsbad, CA, USA). cDNA was synthesized from 500 ng of RNA using the Prime Script RT reagent kit (TaKaRa, Otsu, Shiga, Japan). Real-time qPCR (RT-qPCR) of SDC1 was carried out with the SYBR Prime Script TM RT-PCR kit (TaKaRa), using GAPDH for normalization. The forward and reverse primers were as follows: 5′-CGTGGGGCTCATCTTTGCT-3′ and 5′-TGGCTTGTTTCGGCTCCTC-3′ for SDC1 and 5′-GGTCGTATTGGGCGCCTGGTCACC-3′ and 5′-CACACCCATGACGAACATGGGGGC-3′ for GAPDH. The 2^−ΔΔCt^ method was used to calculate gene expression levels.

### Transwell Migration Assay

A total of 3 × 10^4^ cells in 200 μl of serum-free DMEM were seeded in the upper chamber of a 24-well insert (8 μm, Corning Costar). DMEM with 20% FBS (800 μl) was used as an attractant in the lower chamber. The chambers were incubated in a humidified atmosphere of 5% CO_2_ at 37°C for 48 h. The non-invading cells on the upper surface were removed by gentle scrubbing, and the invading cells on the lower surface were fixed with 4% polyformaldehyde and stained with crystal violet. Then, images of the stained cells were captured by a microscope.

### Statistical Analysis

Statistical analyses were performed using SPSS software (version 24.0). Student's *t*-tests were used to identify significant differences in numeric data. The Kaplan–Meier (KM) method and log rank test were used to analyze the influence of mRNA expression on OS. *P* < 0.05 was regarded as the criterion for a statistically significant difference.

## Results

### Identification of Differentially Expressed Genes

**Figure 1:**
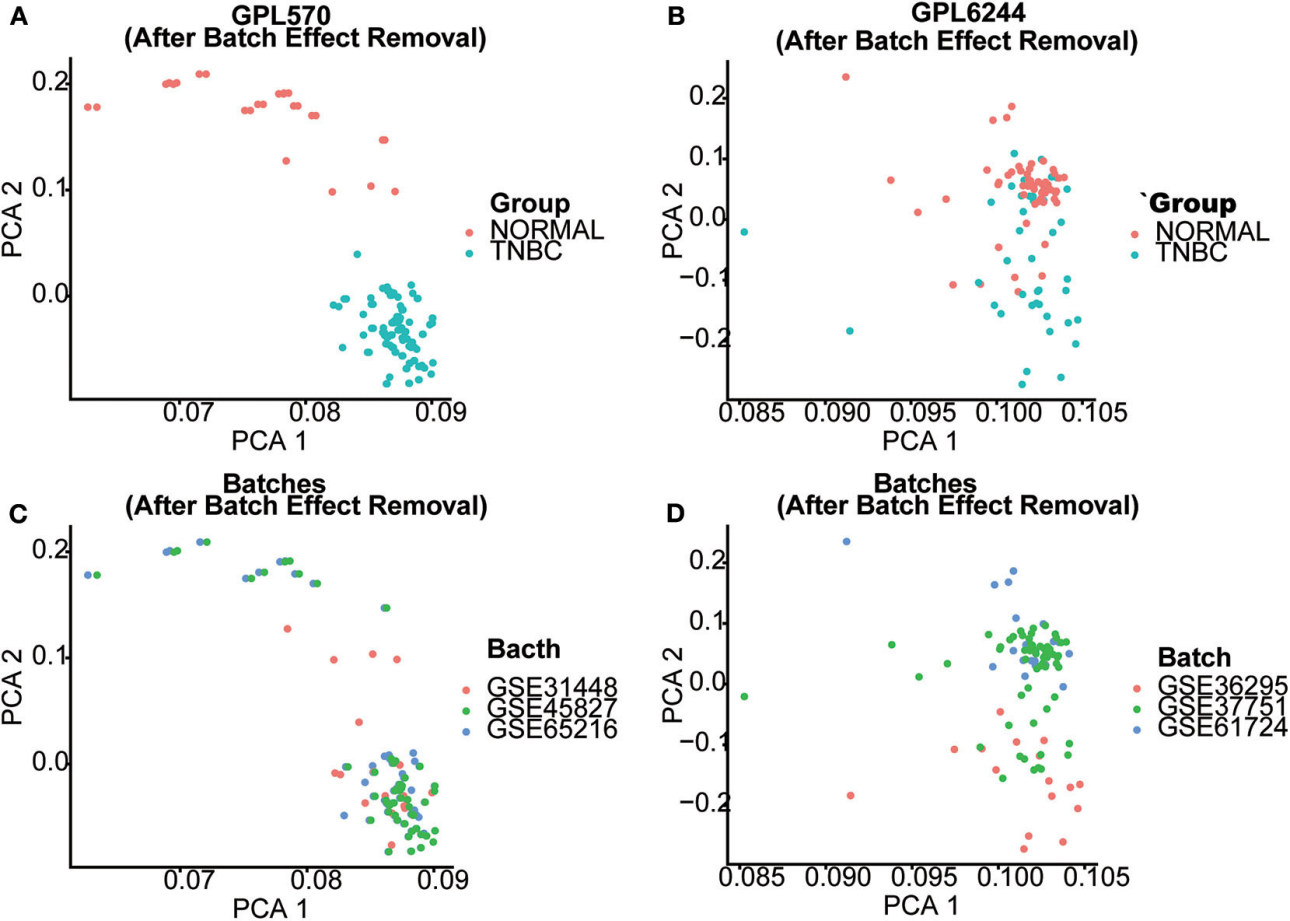
Batch effect correction of GPL570- and GPL6244-based datasets. The effectiveness of batch effect correction is demonstrated using a principal component analysis (PCA) plot. **(A,B)** The PCA plot of GPL570- and GPL6244-based datasets after batch effect correction. Red dots represent normal breast tissues, whereas blue dots represent triple-negative breast cancer (TNBC) samples. **(C,D)** PCA plots of the GPL570- and GPL6244-based datasets after batch effect correction. Samples from different GSE datasets are marked by their colors. After the batch effect correction, samples from different GSE datasets were mixed in the PCA plot, whereas normal breast tissues and TNBC samples were clearly separated from each other. This implies that the batch effect correction process was sufficient without reducing the difference between normal breast tissue and TNBC samples.

**Figure 2:**
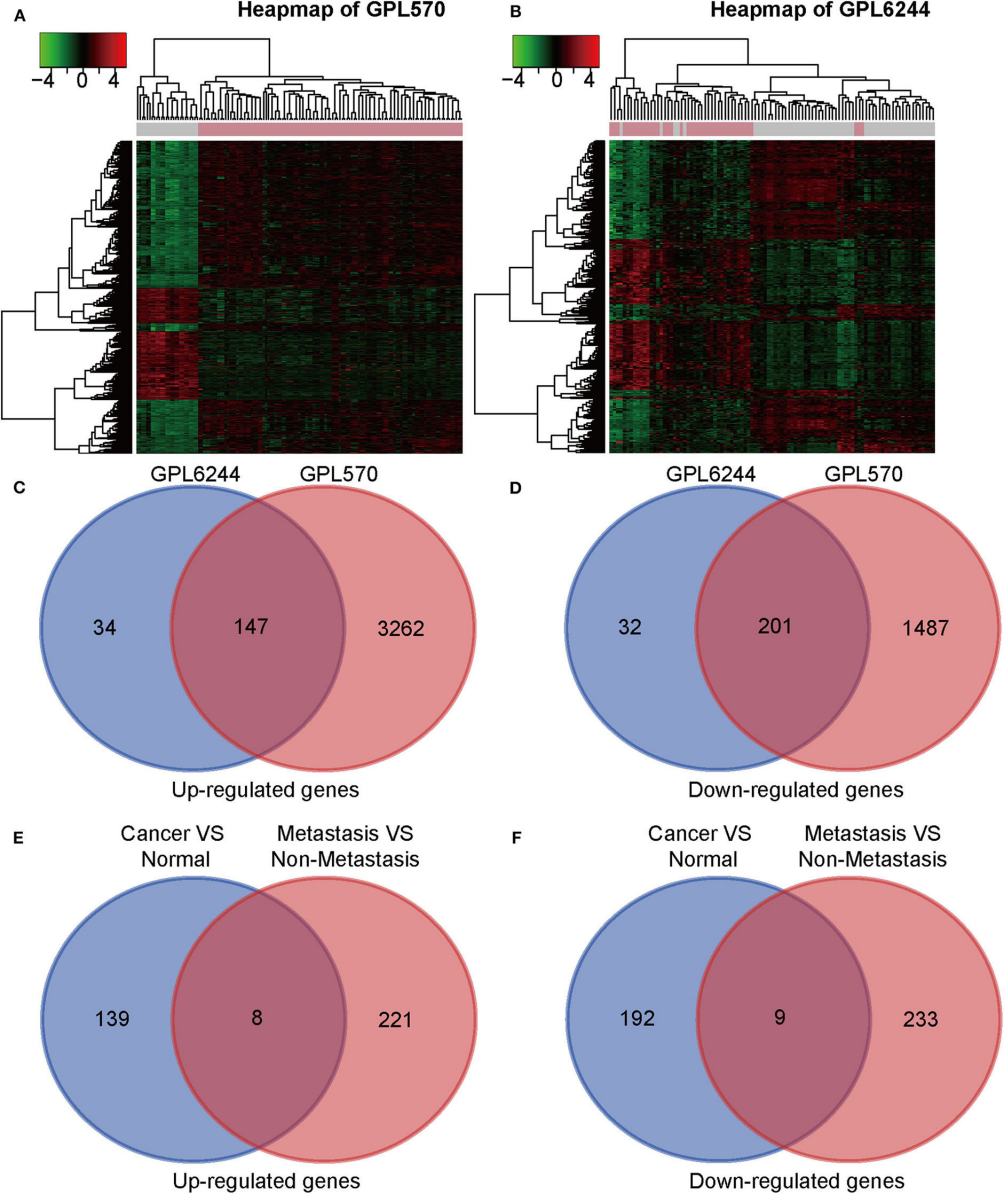
Identification of differentially expressed genes (DEGs). **(A,B)** Heat map of DEGs on the basis of GPL6224 and GPL570 in the integrated microarray analysis. Each column represents one sample, and each row represents one gene. The color spectrum ranging from green to red represents the range of downregulation or upregulation. Each column represents one sample, and each row represents one gene. **(C,D)** Venn diagrams of the upregulated and downregulated DEGs between the datasets based on GPL6224 and GPL570. **(E)** A total of 147 genes were found to be upregulated in triple-negative breast cancer (TNBC) tissues compared with normal breast tissue. A total of 229 genes were found to be upregulated in metastatic TNBC tissues compared with normal breast tissue. With the use of a Venn diagram, eight genes were found to overlap between the two gene sets (*SQLE, RASD2, SDC1, CXADR, SPP1, MMP11, S100A7*, and *S100P*). **(F)** A total of 201 genes were found to be downregulated in TNBC tissues compared with normal breast tissue. A total of 242 genes were found to be downregulated in metastatic TNBC tissues compared with normal breast tissue. With the use of a Venn diagram, nine genes were found to overlap between the two gene sets (*GIPC2, CX3CR1, TPRG1, IGSF10, RBMS3, TGFBR2, RNF125, LIFR*, and *COL6A6*).

Seven gene expression profiles (GSE65216, GSE31448, GSE45827, GSE61724, GSE36295, GSE37751, and GSE103091) were selected in this study. For the profiles from the different batches, batch effect correction was conducted according to the platforms, as shown in Figure 1. Based on the criteria of *P* < 0.01 and |FC| ≥ 1.5, 5,097 DEGs were identified from the profiles based on GPL570, including 3,409 upregulated genes and 1,688 downregulated genes; 414 DEGs were identified from the profiles on the basis of GPL6244, including 181 upregulated genes and 233 downregulated genes. The expression levels of all the genes from the two platforms (5,097 from GPL570 and 414 from GPL6244) are presented in the heatmaps (Figures 2A,B). Subsequently, Venn analysis was performed to obtain the intersection of the DEGs between the two platforms. As show in Figures 2C,D, 147 upregulated genes and 201 downregulated genes overlapped across the six datasets that were based on the two platforms.

DEGs between TNBC with metastasis and without metastasis were obtained from the GEO2R online analysis tool. We identified 229 upregulated DEGs and 242 downregulated DEGs in GSE103091. Then we conducted Venn analysis of the DEGs from GSE103091 and the DEGs from the two platforms above and obtained eight upregulated overlapping DEGs and nine downregulated overlapping DEGs.

### Functional Enrichment Analysis of Overlapping Differentially Expressed Genes

**Figure 3:**
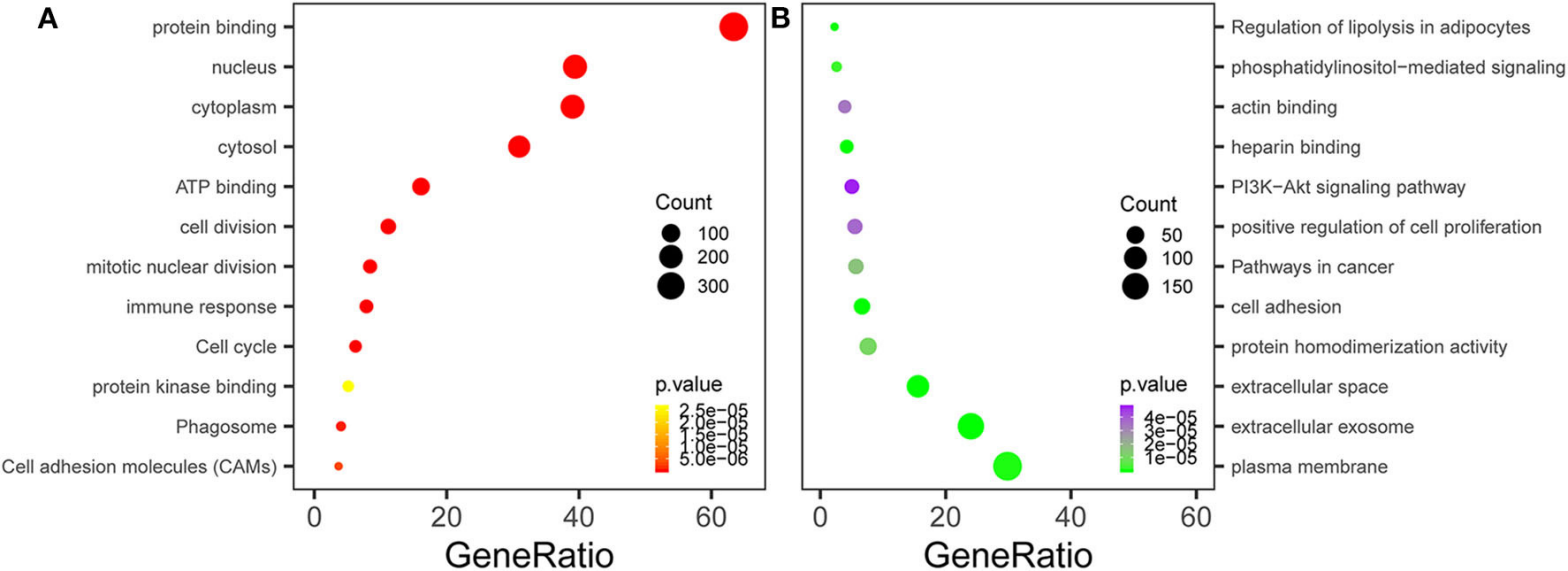
Functional enrichment analysis of the overlapping differentially expressed genes (DEGs). **(A)** Gene Ontology (GO) and Kyoto Encyclopedia of Genes and Genomes (KEGG) enrichment analyses of the overlapping DEGs. The *y*-axis shows significantly enriched GO terms, and the *x*-axis shows the enriched genes to all DEG ratios. Count refers to the number of DEGs enriched in a GO or KEGG term. **(A)** KEGG and GO enrichment of the upregulated overlapping genes. **(B)** KEGG and GO enrichment of the downregulated overlapping genes.

We conducted GO and KEGG pathway enrichment analyses to elucidate the potential biological functions of overlapping genes. The top three enriched GO terms for each category, cell component (CC), BP, and MFs, were identified. For the 147 upregulated genes, the enriched CC terms included the nucleus, cytoplasm, and cytosol. Enriched BP terms included cell division, mitotic nuclear division, and immune response. Enriched MF terms included protein binding, ATP binding, and protein kinase binding. In terms of the 201 downregulated genes, the enriched CC terms included plasma membrane, extracellular exosome, and extracellular space. Enriched BP terms included cell adhesion, positive regulation of cell proliferation, and phosphatidylinositol-mediated signaling. Enriched MF terms included protein homodimerization activity, heparin binding, and actin binding (Table 2, Figure 3A). Regarding KEGG pathway enrichment analysis, the upregulated genes were mainly enriched in the cell cycle, phagosomes, and cell adhesion molecules (CAMs); and the downregulated genes mainly participated in the pathways in cancer, PI3K–Akt signaling pathway, and regulation of lipolysis in adipocytes (Table 2, Figure 3B).

**Table 2:** GO and KEGG enrichment analyses of the overlapping DEGs.

	**Term**	**Description**	**Count**	***P*-value**
Upregulated pathways	KEGG: hsa04110	Cell cycle	34	5.51E−19
	KEGG: hsa04145	Phagosome	22	6.41E−07
	KEGG: hsa04514	Cell adhesion molecules (CAMs)	20	3.28E−06
	GO: 0051301	Cell division	61	3.44E−28
	GO: 0007067	Mitotic nuclear division	46	2.15E−22
	GO: 0006955	Immune response	43	1.60E−11
	GO: 0005634	Nucleus	215	1.19E−07
	GO: 0005737	Cytoplasm	213	1.45E−08
	GO: 0005829	Cytosol	169	3.28E−14
	GO: 0005515	Protein binding	346	3.57E−14
	GO: 0005524	ATP binding	88	9.83E−10
	GO: 0019901	Protein kinase binding	28	2.64E−05
Downregulated pathways	GO: 0007155	Cell adhesion	41	2.65E−08
	GO: 0008284	Positive regulation of cell proliferation	34	3.73E−05
	GO: 0048015	Phosphatidylinositol-mediated signaling	16	2.15E−06
	GO: 0005886	Plasma membrane	184	5.67E−07
	GO: 0070062	Extracellular exosome	148	4.26E−10
	GO: 0005615	Extracellular space	96	1.90E−13
	GO: 0042803	Protein homodimerization activity	47	1.04E−05
	GO: 0008201	Heparin binding	26	5.32E−11
	GO: 0003779	Actin binding	24	3.43E−05
	KEGG: hsa05200	Pathways in cancer	35	1.60E−05
	KEGG: hsa04151	PI3K–Akt signaling pathway	31	4.78E−05
	KEGG: hsa04923	Regulation of lipolysis in adipocytes	14	2.19E−07

*KEGG, Kyoto Encyclopedia of Genes and Genomes; GO, gene ontology; DEGs, differentially expressed genes*.

### Survival Analysis

**Figure 4:**
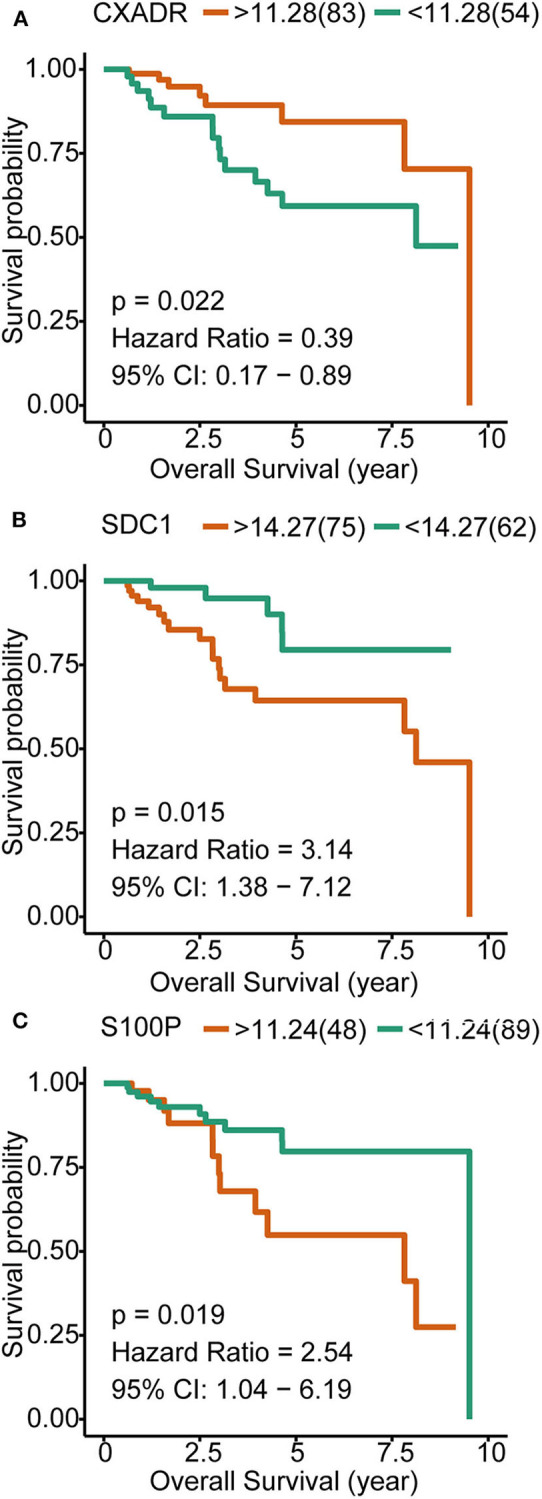
Kaplan–Meier overall survival analyses for the three overlapping genes expressed in triple-negative breast cancer (TNBC) patients. **(A)** Influence of CXADR on OS of TNBC patients. **(B)** Influence of SDC1 on OS of TNBC patients. **(C)** Influence of S100P on OS of TNBC patients.

To investigate the prognostic values of the overlapping genes that correlated with both pathogenesis and metastasis, we analyzed the OS of a total of 138 breast cancer patients with available data in TCGA. A total of 17 overlapping genes (Figures 2E,F) were subjected to survival analysis, three of which were significantly correlated with survival time, including *S100P, SDC1*, and *CXADR. S100P* and *SDC1*, which had Hazard Ratios >1, were identified as poor prognostic genes. Although *CXADR* may be a protective factor according to the survival analysis, it is upregulated in metastatic TNBC and cancer tissue, which suggests the possibility of a false discovery in the survival analysis. Therefore, *CXADR* was not further considered as a biomarker or treatment target (Figure 4).

### SDC1 Knockdown and Overexpression

**Figure 5:**
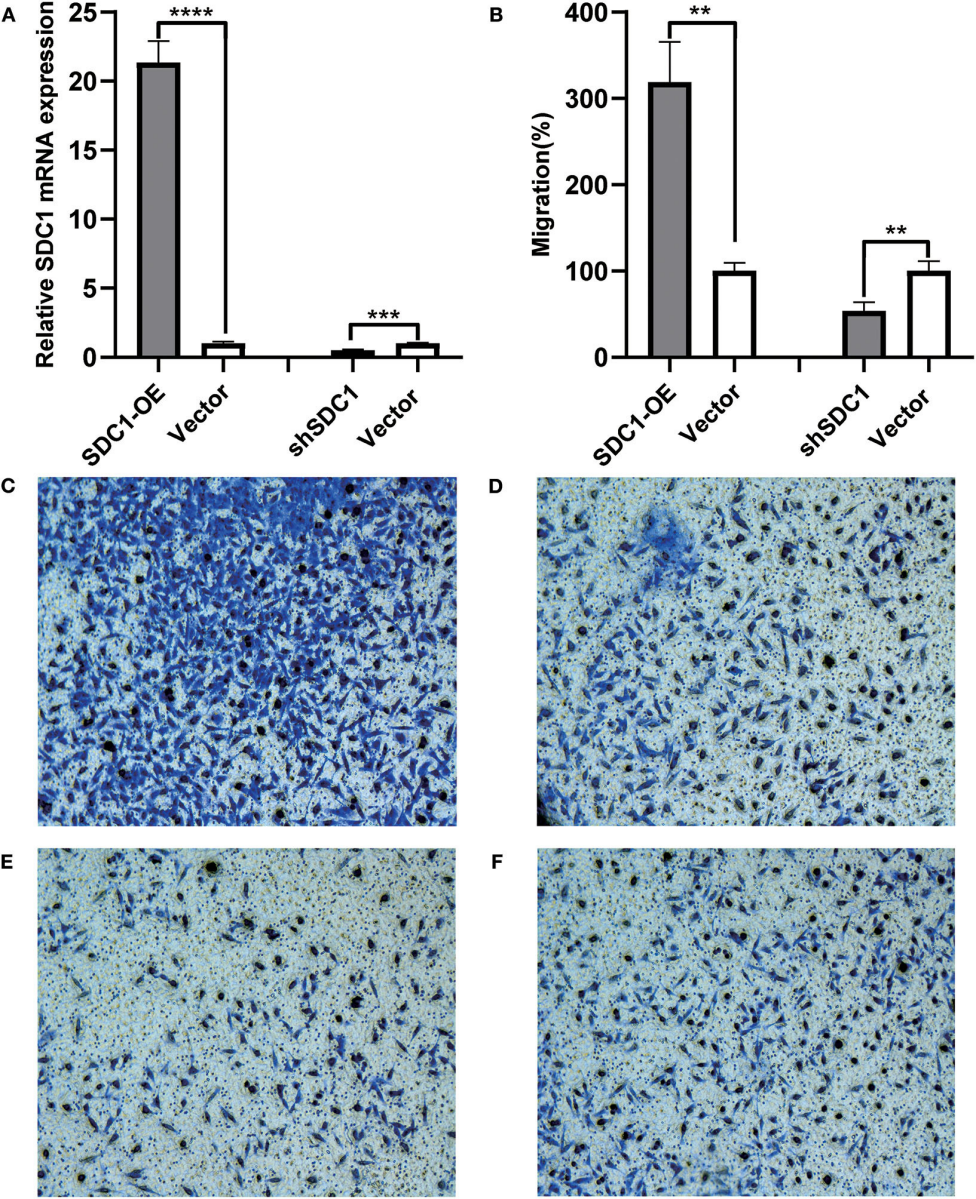
Influence of SDC1 overexpression and knockdown on the migration of triple-negative breast cancer (TNBC) cells. **(A)** The relative mRNA expression of SDC1 was significantly elevated in SDC1-OE-MDA-MB-231 cells, compared with vector-MDA-MB-231 cells (*P* < 0.0001). The relative mRNA expression of SDC1 was significantly reduced in shSDC1-MDA-MB-231 cells, compared with vector-MDA-MB-231 cells (*P* = 0.0006). **(B)** The ratio of migrated cells was significantly elevated in DC1-OE-MDA-MB-231 cells, compared with vector-MDA-MB-231 cells (*P* = 0.0014). The ratio of migrated cells was significantly reduced in shSDC1-MDA-MB-231 cells, compared with vector-MDA-MB-231 cells. (*P* = 0.0061). **(C–F)** The images of bottom surface of upper chambers of SDC1-OE-MDA-MB-231 **(C)**, ShSDC1-MDA-MB-231 **(E)**, and the vector-MDA-MB-231 cells **(D,F)**. Cells were cultured in the upper chamber of the transwell chambers for 48 h and then stained with Diff-Quick dye. **P < 0.01, ***P < 0.001 ****P < 0.0001.

We searched studies on the function of *S100P* and *SDC1* in TNBC and found that the influence of the *S100P* protein in TNBC is already well-defined, but the effect of SDC1 on the invasion of TNBC has not yet been reported. Therefore, we created SDC1-overexpressing MDA-MB-231 cells (SDC1-OE MDA-MB-231) and SDC1-knockdown MDA-MB-231 cells (shSDC1-MDA-MB-231). Further, the mRNA expression of *SDC1* in SDC1-OE-MDA-MB-231, shSDC1-MDA-MB-231, and MDA-MB-231 cells transfected with empty vectors (vector-MDA-MB-231) was validated using RT-PCR (Figure 5A).

### Transwell Migration Assay

To evaluate the influence of SDC1 protein on the invasion of breast cancer cells, transwell migration assay was performed. The SDC1-OE-MDA-MB-231 group showed more migrating cells than did the vector-MDA-MB-231 group (*P* = 0.0014, Figures 5B–D), and the shSDC1-MDA-MB-231 group showed more migrating cells than the vector-MDA-MB-231 group (*P* = 0.0061, Figures 5B,E,F).

## Discussion

In recent years, bioinformatics analysis has been widely used to determine the mechanisms of carcinogenesis, invasion, and metastasis and identify therapeutic targets for various cancers. For example, He et al. identified hub genes related to prostate cancer from GEO datasets and TCGA databases with an integrated method including DEG screening, KEGG pathway analysis, and Protein-protein interaction networks and validated their results with RT-qPCR (10). Sang et al. identified key genes and pathways related to the pathogenesis of HCC by a network-based method that combined data on gene expression and DNA methylation (11). In our previous research, we used the gene expression profiles from the GEO dataset and identified a potential pathway for the mechanism of trastuzumab resistance in HER2-positive breast cancer (12).

Breast cancer is a heterogeneous disease, not only in pathological features but also in molecular characteristics, which causes remarkable difficulty for the treatment of breast cancer. Perou et al. first reported the classification of different subtypes of breast cancer on the basis of variations in gene expression patterns derived from cDNA microarrays (13, 14). Based on the “molecular portrait,” breast tumors can be divided into five large subtypes: normal breast-like, luminal A, luminal B, and HER2-enriched and basal-like breast cancer. TNBC is a unique subtype that lacks ER, PR, and HER2 protein expression. There is ~70–80% concordance between the terms “basal-like” and “triple-negative” (15). Approximately 77% of the basal-like tumors appear as TNBC signatures (16), whereas ~80% of TNBC samples are basal-like (17). Compared with other subtypes of breast cancer, TNBC has a markedly worse prognosis because of the lack of targeted therapies. Hence, it is critical to explore the alteration of the TNBC transcriptome, which is the key to the discovery of new treatment targets.

In the current study, we integrated and analyzed the data of six GEO datasets, including 152 TNBC tissues and 83 normal breast tissues, to identify potential treatment targets for TNBC. A total of 348 DEGs were identified, including 147 upregulated and 201 downregulated genes. The functional enrichment analysis showed that the upregulated genes primarily participated in various cell proliferation processes, including cell division, mitotic nuclear division, protein binding, ATP binding, and protein kinase binding, which was consistent with the biological characteristics of the abnormally rapid proliferation of TNBC cells. The downregulated genes had important functions in the plasma membrane, extracellular exosomes, and in cell adhesion; positive regulation of cell proliferation; protein homodimerization activity; heparin binding; and so on. With respect to the KEGG pathway enrichment analysis, upregulated genes were mostly enriched in pathways related to cell cycle, CAMs, and phagosomes, which suggested that these genes might be important in the proliferation and migration of TNBC cells.

We further explored whether these genes were associated with TNBC metastasis. We found that eight of the 147 upregulated DEGs (*SQLE, RASD2, SDC1, CXADR, SPP1, MMP11, S100A7*, and *S100P*) were also upregulated in TNBC patients with metastasis compared with those without metastasis. Then, using the survival data from the TCGA database, we identified that elevated expression of *S100P* and *SDC1* mRNA is significantly correlated with poor OS of TNBC patients. S100P is a member of the large family of S100 calcium-binding proteins that mediate Ca^2+^-dependent signal transduction pathways, and elevated levels of S100P have been described in a variety of human cancers (18). S100P is reported to induce metastatic phenotype transformation in a benign rat mammary tumor model (19), and S100P overexpression is associated with immortalization of human breast epithelial cells *in vitro* (20). Furthermore, clinical studies showed that a high expression level of S100P protein is significantly associated with a higher rate of recurrence and metastasis, as well as poor survival of TNBC patients (21). The association between S100P protein and poor survival was also reported in breast cancer of all stages (22–24). These studies, together with the high-throughput mRNA data mentioned above, suggest that S100P plays an important role in the carcinogenesis and metastasis of TNBC. S100P has already been considered a treatment target for breast cancer (25).

SDC1 (syndecan-1, CD138) is an abundant heparan sulfate proteoglycan expressed by most epithelial cells (26). SDC1 is a classical coreceptor for growth factors, angiogenic factors, and chemokines and acts as a cell and matrix adhesion receptor (27). A meta-analysis synthesized the data of 1,305 breast cancer patients from nine studies and revealed that high SDC1 expression has been associated with poor prognosis in breast cancer (28). Ibrahim et al. measured the expression of SDC1 mRNA and protein using qPCR and immunohistochemistry (IHC) and found significant elevation of SDC1 expression in triple-negative inflammatory breast cancer (29). Syndecan-1 blockade in the TNBC cell line significantly reduced the 3D spheroid formation and colony formation of TNBC cells (29). Using high-throughput data and qPCR analysis, we also found that SDC1 is upregulated in TNBC, especially in metastatic TNBC. However, the influence of SDC1 on the migration of TNBC cells has not yet been studied. Our transwell migration assay showed that SDC1 overexpression in MDA-MB-231 cells, a TNBC cell line, led to enhanced migration ability as compared with that in the control cells. When SDC1 expression was blocked by shRNA, the migration of MDA-MB-231 cells was markedly attenuated. Aside from the influence on the migration ability of TNBC cells, SDC1 knockdown was also found to reduce the lung metastatic burden by over 97% in a lung metastasis mouse model (30). Silencing the expression of SDC1 in TNBC cells significantly reduced metastasis to the brain, and conversely, overexpression of SDC1 increased metastasis to the brain (31). Together, these findings demonstrate the critical role of SDC1 in the proliferation, migration, and metastasis of TNBC. Therefore, SDC1 could be used as a potential treatment target and prognosis biomarker for TNBC.

## Conclusion

In conclusion, using integrated bioinformatics analysis, we identified that S100P and SDC1 were upregulated in metastatic TNBC and that SDC1 elevation in TNBC tissues may suggest a poor OS. Further, we found that SDC1 overexpression augmented the migration ability of TNBC cells and that SDC1 blockade remarkably attenuated the migration of TNBC cells. The role of S100P in the carcinogenesis and metastasis of TNBC has already been well-defined. In conclusion, S100P and SDC1 may be potential treatment targets as well as prognostic biomarkers of TNBC.

## Data Availability Statement

Publicly available datasets were analyzed in this study. This data can be found here: The Cancer Genome Atlas (https://portal.gdc.cancer.gov/) (TCGA BLCA dataset) and the Gene Expression Omnibus (GEO, https://www.ncbi.nlm.nih.gov/geo/) (GSE65216, GSE31448, GSE45827, GSE61724, GSE36295, GSE37751, and GSE103091).

## Ethics Statement

All data used in this study came from the GEO database, and no clinical trial or animal experiments were included in this study. This study was granted an exemption from requiring ethical approval by the ethics committee of Beijing Union Hospital (Beijing, China).

## Author Contributions

QS and SS: data analysis and study design. YZ and YX: bioinformatics analysis. BZ: cell culture, miRNA blockade, and migration assay.

## Conflict of Interest

The authors declare that the research was conducted in the absence of any commercial or financial relationships that could be construed as a potential conflict of interest.

## References

[1] Siegel RL Miller KD Jemal A Cancer statistics, 2018. CA Cancer J Clin. (2018) 68:7–30. 10.3322/caac.21442 29313949

[2] Cancer Genome Atlas N Comprehensive molecular portraits of human breast tumours. Nature. (2012) 490:61–70. 10.1038/nature11412 23000897 PMC3465532

[3] Kohler BA Sherman RL Howlader N Jemal A Ryerson AB Henry KA. Annual report to the nation on the status of cancer, 1975-2011, featuring incidence of breast cancer subtypes by race/ethnicity, poverty, and state. J Natl Cancer Inst. (2015) 107:djv048. 10.1093/jnci/djv048 25825511 PMC4603551

[4] Lehmann BD Bauer JA Chen X Sanders ME Chakravarthy AB Shyr Y. Identification of human triple-negative breast cancer subtypes and preclinical models for selection of targeted therapies. J Clin Invest. (2011) 121:2750–67. 10.1172/JCI45014 21633166 PMC3127435

[5] Fornier M Fumoleau P. The paradox of triple negative breast cancer: novel approaches to treatment. Breast J. (2012) 18:41–51. 10.1111/j.1524-4741.2011.01175.x 22098334

[6] Kulasingam V Diamandis EP. Strategies for discovering novel cancer biomarkers through utilization of emerging technologies. Nat Clin Pract Oncol. (2008) 5:588–99. 10.1038/ncponc1187 18695711

[7] Li MX Jin LT Wang TJ Feng YJ Pan CP Zhao DM. Identification of potential core genes in triple negative breast cancer using bioinformatics analysis. Onco Targets Ther. (2018) 11:4105–12. 10.2147/OTT.S166567 30140156 PMC6054764

[8] Liu X Wu J Zhang D Bing Z Tian J Ni M. Identification of potential key genes associated with the pathogenesis and prognosis of gastric cancer based on integrated bioinformatics analysis. Front Genet. (2018) 9:265. 10.3389/fgene.2018.00265 30065754 PMC6056647

[9] Zeng F Fu J Hu F Tang Y Fang X Zeng F. Identification of key pathways and genes in response to trastuzumab treatment in breast cancer using bioinformatics analysis. Oncotarget. (2018) 9:32149–60. 10.18632/oncotarget.24605 30181805 PMC6114942

[10] He Z Tang F Lu Z Huang Y Lei H Li Z. Analysis of differentially expressed genes, clinical value and biological pathways in prostate cancer. Am J Transl Res. (2018) 10:1444–56. 29887958 PMC5992552

[11] Sang L Wang XM Xu DY Zhao WJ. Bioinformatics analysis of aberrantly methylated-differentially expressed genes and pathways in hepatocellular carcinoma. World J Gastroenterol. (2018) 24:2605–16. 10.3748/wjg.v24.i24.2605 29962817 PMC6021769

[12] Zhao B Zhao Y Sun Y Niu H Sheng L Huang D. Alterations in mRNA profiles of trastuzumabresistant Her2positive breast cancer. Mol Med Rep. (2018) 18:139–46. 10.3892/mmr.2018.8981 29750305 PMC6059662

[13] Perou CM Sorlie T Eisen MB van de Rijn M Jeffrey SS Rees CA. Molecular portraits of human breast tumours. Nature. (2000) 406:747–52. 10.1038/35021093 10963602

[14] Sorlie T Perou CM Tibshirani R Aas T Geisler S Johnsen H. Gene expression patterns of breast carcinomas distinguish tumor subclasses with clinical implications. Proc Natl Acad Sci USA. (2001) 98:10869–74. 10.1073/pnas.191367098 11553815 PMC58566

[15] Diana A Franzese E Centonze S Carlino F Corte CMD Ventriglia J. Triple-negative breast cancers: systematic review of the literature on molecular and clinical features with a focus on treatment with innovative drugs. Curr Oncol Rep. (2018) 20:76. 10.1007/s11912-018-0726-6 30128845

[16] Sporikova Z Koudelakova V Trojanec R Hajduch M. Genetic markers in triple-negative breast cancer. Clin Breast Cancer. (2018) 18:e841–50. 10.1016/j.clbc.2018.07.023 30146351

[17] Temian DC Pop LA Irimie AI Berindan-Neagoe I. The epigenetics of triple-negative and basal-like breast cancer: current knowledge. J Breast Cancer. (2018) 21:233–43. 10.4048/jbc.2018.21.e41 30275851 PMC6158152

[18] Prica F Radon T Cheng Y Crnogorac-Jurcevic T. The life and works of S100P - from conception to cancer. Am J Cancer Res. (2016) 6:562–76. 27186425 PMC4859681

[19] Wang G Platt-Higgins A Carroll J de Silva Rudland S Winstanley J Barraclough R. Induction of metastasis by S100P in a rat mammary model and its association with poor survival of breast cancer patients. Cancer Res. (2006) 66:1199–207. 10.1158/0008-5472.CAN-05-2605 16424059

[20] Guerreiro Da Silva ID Hu YF Russo IH Ao X Salicioni AM Yang X. S100P calcium-binding protein overexpression is associated with immortalization of human breast epithelial cells *in vitro* and early stages of breast cancer development *in vivo*. Int J Oncol. (2000) 16:231–40. 10.3892/ijo.16.2.231 10639564

[21] Maierthaler M Kriegsmann M Peng C Jauch S Szabo A Wallwiener M. S100P and HYAL2 as prognostic markers for patients with triple-negative breast cancer. Exp Mol Pathol. (2015) 99:180–7. 10.1016/j.yexmp.2015.06.010 26112095

[22] Maciejczyk A Lacko A Ekiert M Jagoda E Wysocka T Matkowski R. Elevated nuclear S100P expression is associated with poor survival in early breast cancer patients. Histol Histopathol. (2013) 28:513–24. 10.14670/HH-28.513 23364898

[23] Chung L Phillips L Lin MZ Moore K Marsh DJ Boyle FM. A novel truncated form of S100P predicts disease-free survival in patients with lymph node positive breast cancer. Cancer Lett. (2015) 368:64–70. 10.1016/j.canlet.2015.07.046 26276712

[24] Peng C Chen H Wallwiener M Modugno C Cuk K Madhavan D. Plasma S100P level as a novel prognostic marker of metastatic breast cancer. Breast Cancer Res Treat. (2016) 157:329–38. 10.1007/s10549-016-3776-1 27146585

[25] Arumugam T Logsdon CD. S100P: a novel therapeutic target for cancer. Amino Acids. (2011) 41:893–9. 10.1007/s00726-010-0496-4 20509035 PMC4041611

[26] McDermott SP Ranheim EA Leatherberry VS Khwaja SS Klos KS Alexander CM. Juvenile syndecan-1 null mice are protected from carcinogen-induced tumor development. Oncogene. (2007) 26:1407–16. 10.1038/sj.onc.1209930 16953225

[27] Nikolova V Koo CY Ibrahim SA Wang Z Spillmann D Dreier R. Differential roles for membrane-bound and soluble syndecan-1 (CD138) in breast cancer progression. Carcinogenesis. (2009) 30:397–407. 10.1093/carcin/bgp001 19126645

[28] Qiao W Liu H Guo W Li P Deng M. Prognostic and clinical significance of syndecan-1 expression in breast cancer: a systematic review and meta-analysis. Eur J Surg Oncol. (2019) 45:1132–7. 10.1016/j.ejso.2018.12.019 30598194

[29] Ibrahim SA Gadalla R El-Ghonaimy EA Samir O Mohamed HT Hassan H. Syndecan-1 is a novel molecular marker for triple negative inflammatory breast cancer and modulates the cancer stem cell phenotype via the IL-6/STAT3, Notch and EGFR signaling pathways. Mol Cancer. (2017) 16:57. 10.1186/s12943-017-0621-z 28270211 PMC5341174

[30] Chute C Yang X Meyer K Yang N O'Neil K Kasza I. Syndecan-1 induction in lung microenvironment supports the establishment of breast tumor metastases. Breast Cancer Res. (2018) 20:66. 10.1186/s13058-018-0995-x 29976229 PMC6034333

[31] Sayyad MR Puchalapalli M Vergara NG Wangensteen SM Moore M Mu L. Syndecan-1 facilitates breast cancer metastasis to the brain. Breast Cancer Res Treat. (2019) 178:35–49. 10.1007/s10549-019-05347-0 31327090

